# Association of Cholecystectomy with Metabolic Syndrome in a Chinese Population

**DOI:** 10.1371/journal.pone.0088189

**Published:** 2014-02-05

**Authors:** Chao Shen, Xiaoliang Wu, Chengfu Xu, Chaohui Yu, Peng Chen, Youming Li

**Affiliations:** 1 International Health Care Center, the First Affiliated Hospital, College of Medicine, Zhejiang University, Hangzhou, Zhejiang, China; 2 Intensive Care Unit, the First Affiliated Hospital, College of Medicine, Zhejiang University, Hangzhou, Zhejiang, China; 3 Department of Gastroenterology, the First Affiliated Hospital, College of Medicine, Zhejiang University, Hangzhou, Zhejiang, China; National Institute for Viral Disease Control and Prevention, CDC, China

## Abstract

An association between cholecystectomy and metabolic syndrome has not been fully established. Here we analyzed the association between cholecystectomy and metabolic syndrome in a Chinese population of 5672 subjects who undergone annual health checkups at the First Affiliated Hospital, College of Medicine, Zhejiang University between January 2011 and December 2012. The prevalences of gallstones, cholecystectomy and metabolic syndrome were 6.0%, 3.6%, and 32.5%, respectively. The prevalence of metabolic syndrome was significantly higher in subjects with a history of cholecystectomy (63.5%) than in those with gallstones (47.0%) or in those without gallstone disease (30.3%; *P*<0.01 for both). Multivariate logistic regression analysis showed that cholecystectomy was significantly associated with increased risk of metabolic syndrome (OR = 1.872; 95% CI: 1.193–2.937). However, the association of gallstones with metabolic syndrome was not statistically significant (OR = 1.267; 95% CI: 0.901–1.782). Altogether, our results suggest that cholecystectomy significantly increases the risk of metabolic syndrome.

## Introduction

Metabolic syndrome is a cluster of disorders that include abdominal obesity, dyslipidemia, hypertension, and impaired glucose tolerance [1], [2]. Epidemiological studies showed that more than one-third of adults and a rapidly increasing number of children have metabolic syndrome in the United States [3], [4], and it is epidemic in developing countries as well [5], [6]. Metabolic syndrome has been associated with the development of cardiovascular diseases, type 2 diabetes, chronic kidney disease, and nonalcoholic fatty liver disease [7]–[9]. Therefore, metabolic syndrome is a major health concern worldwide.

Obesity is the most important risk factor for metabolic syndrome, and a link between insulin resistance and metabolic syndrome has been established [10], [11]. Nevertheless, despite numerous studies, the risk factors for metabolic syndrome remain insufficiently understood. Previous studies reported that subjects with gallstone diseases were more likely to have metabolic syndrome than those without gallstone disease [12], [13], and patients with complicated gallstone disease had a higher prevalence of metabolic syndrome than those with uncomplicated gallstone disease [14]. Conversely, insulin resistance increases the risk of gallstone disease [15]. These studies point toward an association between gallstone diseases and metabolic syndrome.

Gallstone diseases may be asymptomatic or symptomatic, and the latter often requires cholecystectomy. Several lines of evidence indicate that cholecystectomy may increase risk of metabolic syndrome. For example, serum and hepatic triglyceride levels increased 25% after cholecystectomy in mice [16]. In clinical settings, cholecystectomized patients three years after the surgery were found to have a significant increase in very low density lipoprotein and intermediate density lipoprotein apolipoprotein B (ApoB) concentration [17]. In other studies, a slight deterioration of postprandial glycemic control, increases in body mass index, and risks for cardiovascular disease were observed in patients after cholecystectomy [18]–[21]. Most significantly for the present work, a large sample study reported in 2013 that cholecystectomy, but not gallstones, was associated with nonalcoholic fatty liver disease, a hepatic manifestation of metabolic syndrome [22]. To date, however, the association between cholecystectomy and metabolic syndrome has not been fully defined.

In this study, we performed a large sample, cross-sectional survey to investigate the association between cholecystectomy and metabolic syndrome in a Chinese population.

## Materials and Methods

### Ethics Statement

All participants were informed about the purpose and general procedures of the examination. Written informed consent was not required because of the observational nature of the investigation. The subject information was anonymized at collection and anonymized prior to analysis. The Ethics Committee of the First Affiliated Hospital, College of Medicine, Zhejiang University approved the study’s protocol and manner of consent.

### Subjects

Study subjects were recruited from the participants who had voluntarily undergone annual health checkups at the First Affiliated Hospital, College of Medicine, Zhejiang University between January 2011 and December 2012. The analyses were limited to the subjects who underwent abdominal ultrasonography, and those who had full records of anthropometric and biochemical data. A total of 5672 subjects (3699 men and 1973 women) were included in the final analysis.

### Clinical Examination

Clinical examinations were performed by trained staffs using standardized procedures [23], [24]. Height and weight were measured using an automatic digital stadiometer, with the subjects dressed in a light gown and standing barefoot. Body mass index was calculated as weight in kilograms divided by height in meters squared. Waist circumference was measured using a non-stretchable standard tape. Blood pressure was measured using standardized procedures. Fasting whole blood samples were obtained from an antecubital vein and serum samples were separated for biochemical analysis without freezing. The biochemical values were measured with a Hitachi 7600 autoanalyzer (Hitachi, Tokyo, Japan) using standard methods. Abdominal ultrasonography was performed in each subjects and gallstone disease was defined as ultrasound documented gallstones or evidence of a cholecystectomy. The reason for cholecystectomy was inquired and cholecystectomy due to reasons other than symptomatic gallstones or gallstone-related complications was excluded from the analysis in this study.

### Diagnosis of Metabolic Syndrome

Metabolic syndrome was diagnosed in accordance with the criteria established by the International Diabetes Federation published in 2006 [25]. For a subject who had central obesity (defined for ethnic Chinese as waist circumference ≥90 cm for men and ≥80 cm for women), metabolic syndrome was diagnosed if any two or more of the following were present: (i) triglyceride ≥1.7 mmol/L or specific treatment for this lipid abnormality; (ii) high-density lipoprotein cholesterol <1.03 mmol/L in men and <1.29 mmol/L in women; (iii) systolic blood pressure ≥130 mmHg or diastolic blood pressure ≥85 mmHg, or treatment of previously diagnosed hypertension; or (iv) fasting blood glucose ≥5.6 mmol/L, or previously diagnosed type 2 diabetes.

### Statistical Analyses

Statistical analyses were performed using SPSS 13.0 software for Windows (SPSS Inc., Chicago, IL). Continuous variables were expressed as mean and standard deviation or median and interquartile range (25%–75%), according to the normality of the data. Comparisons between the independent groups were conducted using Student’s *t*-test or the Mann-Whitney *U* test. Categorical variables were compared using the chi-square test. Univariate and multivariate logistic regression analyses were conducted to assess the odds ratio (OR) for metabolic syndrome, comparing subjects with gallstone disease to those without gallstone disease. *P*<0.05 (2-tailed) was considered statistically significant.

## Results

Of 5672 subjects enrolled in this study, 338 had gallstone, and 203 subjects had a history of cholecystectomy (Table 1). Subjects with gallstone diseases were associated with older age and had more metabolic abnormalities than those without gallstone diseases. In comparison to the subjects with gallstone, cholecystectomized subjects were associated with higher body mass index, and waist circumference, and higher levels of alanine aminotransferase, aspartate aminotransferase, triglyceride, and fasting blood glucose. These results indicate that cholecystectomized subjects were significantly more likely to have more metabolic abnormalities than those with gallstones.

**Table 1:** Demographics and clinical characteristics of the subjects according to gallstone disease status.

	All subjects, without or with gallstone disease			Subjects with gallstone diseases		
	Without^a^	With^b^	*P value*	Gallstones^c^	Cholecystectomy^d^	*P value*
Age (years)	50.3 (9.8)	55.0 (10.0)	<0.001	54.8 (10.4)	55.4 (9.2)	0.452
Gender (male/female)	3353/1778	346/195	0.537^e^	230/108	116/87	0.012^e^
Body mass index (kg/m^2^)	24.53 (3.10)	25.56 (3.07)	<0.001	25.28 (3.09)	26.02 (2.98)	0.006
Waist circumference (cm)	86.1 (9.5)	89.4 (9.2)	<0.001	88.8 (9.3)	90.5 (8.9)	0.031
Systolic blood pressure (mmHg)	129.9 (17.6)	135.4 (18.0)	<0.001	134.5 (17.8)	136.9 (18.2)	0.124
Diastolic blood pressure (mmHg)	78.9 (11.4)	82.2 (10.6)	<0.001	82.1 (10.5)	82.5 (10.8)	0.648
Alanine aminotransferase (U/L)	21.0 (15.0–31.0)	23.0 (17.0–34.0)	<0.001^f^	22.0 (15.0–32.0)	26.0 (19.0–38.0)	<0.001^f^
Aspartate aminotransferase (U/L)	21.0 (18.0–25.0)	21.0 (18.0–26.0)	0.093^f^	21.0 (18.0–25.0)	23.0 (19.0–27.0)	0.009^f^
γ-Glutamyltransferase (U/L)	26.0 (16.0–46.0)	30.0 (19.0–55.0)	<0.001^f^	31.0 (19.0–56.0)	30.0 (19.0–27.0)	0.980^f^
Triglyceride (mmol/L)	1.38 (0.96–2.05)	1.69 (1.12–2.46)	<0.001^f^	1.54 (1.05–2.35)	1.88 (1.33–2.55)	0.001^f^
Total cholesterol (mmol/L)	4.93 (0.96)	5.01 (0.96)	0.063	4.99 (0.93)	5.04 (1.01)	0.627
HDL cholesterol (mmol/L)	1.15 (0.30)	1.11 (0.31)	0.004	1.12 (0.30)	1.09 (0.32)	0.156
Fasting blood glucose (mmol/L)	4.73 (4.41–5.13)	4.98 (4.54–5.51)	<0.001^f^	4.92 (4.50–5.28)	5.10 (4.61–6.01)	<0.001^f^
Serum uric acid (μmol/L)	344.2 (88.4)	363.0 (94.9)	<0.001	365.4 (95.8)	358.9 (93.5)	0.440
Metabolic syndrome (Yes/No)	1553/3578	288/253	<0.001	159/179	129/74	<0.001

The data are expressed as the mean ± SD or median (IQR) depending on the data distribution. HDL, high-density lipoprotein.

a n = 5131;

b n = 541;

c n = 338;

d n = 203;

e *χ* ^2^ value;

f *z* value.

The overall prevalence of metabolic syndrome was 32.5%. The prevalence of metabolic syndrome was significantly higher for the subjects with gallstone diseases (53.2%) than those without gallstone diseases (30.3%; *χ* ^2^ test, *P*<0.001). A finding of note was that cholecystectomized subjects were associated with a significantly higher prevalence of metabolic syndrome (63.5%) than those with gallstones (47.0%; *χ* ^2^ test, *P*<0.001).

**Figure 1:**
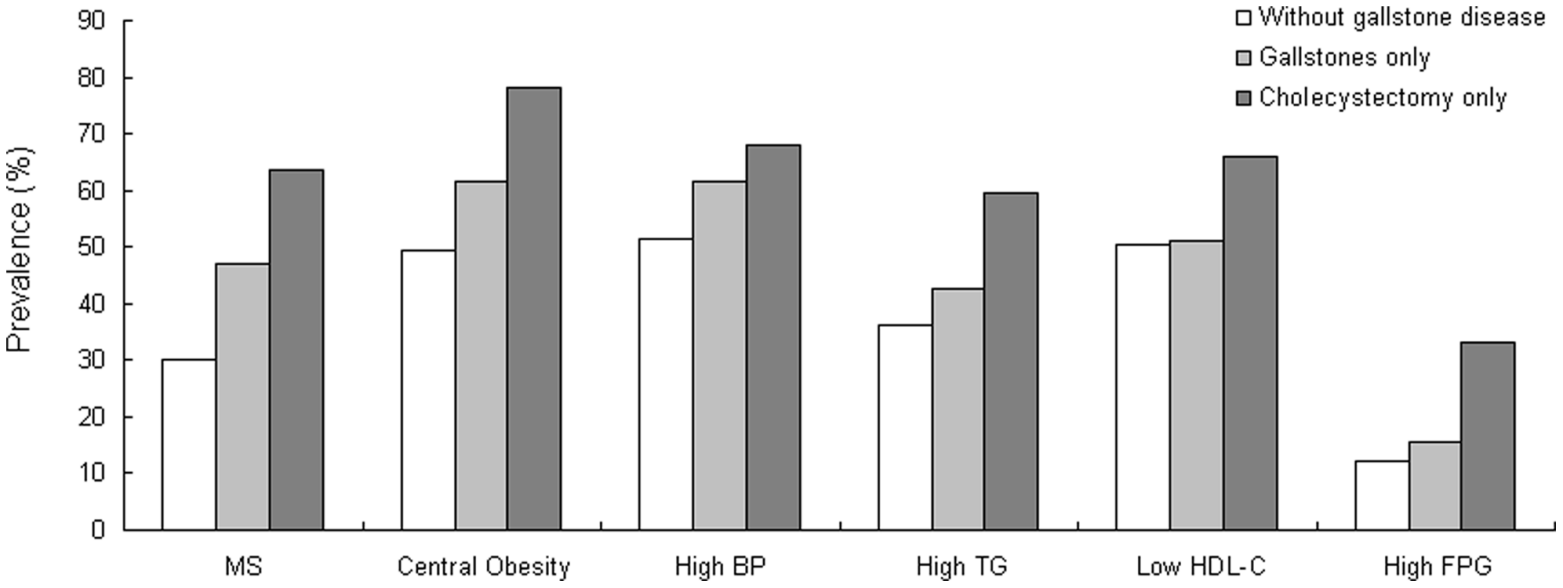
Prevalences of metabolic syndrome and each of its component features. The prevalences of metabolic syndrome and each of its component features in individuals with gallstones were higher than the prevalences in individuals without gallstones, and higher still in those who had undergone cholecystectomy (all with *P*_for trend_ <0.001). BP, blood pressure; TG, triglyceride; HDL-C high-density lipoprotein cholesterol; FPG, fasting blood glucose.

The associations between gallstone diseases and the prevalence of metabolic syndrome compound features were also analyzed (Figure 1). In comparison to the subjects without gallstone, the prevalence of central obesity, elevated triglyceride, low high-density lipoprotein cholesterol, elevated blood pressure, and elevated fasting blood glucose all tended to increase in subjects with gallstones, and further increase in cholecystectomized subjects. These results support a significant association between gallstone diseases, and especially cholecystectomy, with metabolic syndrome.

Logistic regression analysis was performed to investigate the ORs for metabolic syndrome with gallstone diseases (Table 2). Univariate regression analysis showed that both gallstones (OR = 1.929, 95% CI: 1.546–2.406) and cholecystectomy (OR = 3.826, 95% CI: 2.858–5.120) were associated with the risk factors for metabolic syndrome. After adjusting for factors associated with metabolic syndrome, multivariate regression analysis showed that cholecystectomy remains significantly associated with metabolic syndrome (OR = 1.872; 95% CI: 1.193–2.937), while the association of gallstones with metabolic syndrome was not statistically significant (OR = 1.267; 95% CI: 0.901–1.782). These results indicate a stronger association between cholecystectomy and metabolic syndrome than between gallstones and metabolic syndrome.

**Table 2:** Logistic regression analysis of odds ratios for the metabolic syndrome relative to gallstone diseases.

	Univariate regression analysis			Multivariate regression analysis*		
	OR	95% CI	*P value*	OR	95% CI	*P value*
Without gallstone disease	0.381	0.319–0.456	<0.001	0.667	0.504–0.882	0.005
Gallstones only	1.929	1.546–2.406	<0.001	1.267	0.901–1.782	0.173
Cholecystectomy only	3.826	2.858–5.120	<0.001	1.872	1.193–2.937	0.006

* Data are adjusted for age, gender, body mass index, waist circumference, systolic blood pressure, diastolic blood pressure, alanine aminotransferase, aspartate aminotransferase, γ-glutamyltransferase, triglyceride, total cholesterol, high-density lipoprotein cholesterol, fasting blood glucose, and serum uric acid.

## Discussion

In this study, our results indicated that cholecystectomy is significantly associated with metabolic syndrome. Cholecystectomized subjects had a higher prevalence of metabolic syndrome and its compound of features than subjects with gallstones or normal gallbladder. Multivariate regression analysis revealed that cholecystectomy, but not gallstones, was significantly associated with metabolic syndrome. This indicates that metabolic syndrome is more strongly related to cholecystectomy than its relation with gallstones.

The association between gallstone disease and metabolic syndrome has received much attention. Mendez-Sanchez *et al.* [12] were the first report that gallstone disease was strongly associated with metabolic syndrome. Their observation was confirmed by another two large sample studies [13], [26]. Recently, Ata *et al.* [14] reported that metabolic syndrome was more prevalent among subjects with complicated gallstone disease than among those with uncomplicated gallstone disease, suggesting an association between metabolic syndrome and the severity of gallstone disease. Most of these studies only included gallstones, whereas a few have examined cholecystectomy and gallstones separately in regard to metabolic syndrome. Here, we provide evidence that cholecystectomy may be more strongly related to metabolic syndrome than are gallstones.

The underlying reasons that might explain why cholecystectomy showed a stronger association with metabolic syndrome than did gallstone remain uncertain. Logically, the mechanism might involve the alteration of glucose and lipid metabolism caused by loss of the gallbladder. After cholecystectomy, bile is continuously secreted into the duodenal lumen, and 24-hour bile acid output is higher than in health individuals [27]. Bile acids may act as hormonal signals through interactions with several enterohepatic and peripheral target receptors such as farnesoid X receptor (FXR) and bile acid receptor TGR5. Both FXR and TGR5 have crucial roles in the regulation of lipid and glucose metabolism [28], [29]. Another mechanism for that could contribute to the effect of cholecystectomy on the development of metabolic syndrome may be the state of chronic inflammation caused by cholecystectomy. It has been reported that dilation of the common bile duct and increases of bile duct pressure after cholecystectomy causes chronic inflammation in the surrounding liver tissue [30]. Such increased inflammation stimulates the release of cytokines, chemokines, reactive oxygen species, and reactive nitrogen intermediates, all of which may lead to the onset of metabolic syndrome [31], [32]. However, the detailed mechanism requires further investigation.

Several limitations are acknowledged in this study. First, the study population was recruited from individuals who attended a health screening at one university hospital and therefore may not represent the entire community. The second limitation is that the causal relationship between cholecystectomy and metabolic syndrome could not be examined by this cross-sectional study. Finally, information regarding smoking status, physical activity, or alcohol consumption was not available. These factors may act as confounding variables in the association between cholecystectomy and metabolic syndrome. Further longitudinal studies should consider these confounding factors.

Despite the limitations described above, our results provide evidence for the first time that metabolic syndrome is common among persons with gallstone disease, and cholecystectomy showed a stronger association with metabolic syndrome than gallstones. These observations suggest that cholecystectomy significantly increases the risk of metabolic syndrome.
